# Effects of Dietary Calcium and Magnesium Levels on the Growth Performance, Tissue Mineral Deposition, Exoskeleton Development, and Molting Performance of Chinese Mitten Crab (*Eriocheir sinensis*)

**DOI:** 10.1155/anu/4186013

**Published:** 2026-02-17

**Authors:** Han Chen, WenBin Liu, YueYun Guo, SiSi Xiong, ZiShang Liu, YanZou Dong, BeiLe Ye, Lei Xu, Pan Wang, XiangFei Li

**Affiliations:** ^1^ Key Laboratory of Aquatic Nutrition and Feed Science of Jiangsu Province, College of Animal Science and Technology, Nanjing Agricultural University, Nanjing, Jiangsu, 210095, China, njau.edu.cn; ^2^ Key Laboratory of Aquatic Functional Feed and Environmental Regulation of Fujian Province, Fujian DBN Aquatic Sci. and Tech. Co., Ltd, Zhangzhou, Fujian, 363500, China

**Keywords:** calcium–magnesium interaction, *Eriocheir sinensis*, exoskeleton development, molting, tissue mineral deposition

## Abstract

Calcium and magnesium are essential mineral elements for animal growth and development, playing crucial roles in skeletal formation, particularly in the molting process of crustaceans. However, their interaction is still poorly elucidated. This study investigated the effects of dietary calcium and magnesium levels on the growth performance, tissue deposition of calcium and magnesium, exoskeleton development, and molting performance of Chinese mitten crab (*Eriocheir sinensis*). A 2 × 4 factorial design was adopted to formulate eight experimental diets, comprising two targeted calcium levels (1% and 2%) and four targeted magnesium levels (0.15%, 0.3%, 0.45%, and 0.6%). These diets were fed to crabs with an initial body weight of 40.46 ± 0.47 g for a 10‐week period. The results showed that increased dietary calcium levels significantly reduced the weight gain rate (WGR), hepatosomatic index (HSI), carapace hardness, tissue (hepatopancreas, intestine, and muscle) calcium content, the expressions of genes related to calcium and magnesium absorption in the hepatopancreas, and the expressions of genes associated with molting and exoskeleton development. In contrast, it decreased the levels of molting hormone in hemolymph, chitinase (CHI) activity in the epidermis, and the expressions of genes encoding the molting hormone receptor and CHI in the hepatopancreas. The highest WGR was observed at a magnesium level of 0.6%, which also significantly enhanced carapace hardness and methyl farnesoate (MF) content in hemolymph, but significantly suppressed the expressions of chitin synthase genes in the hepatopancreas. Moreover, at 1% calcium level groups, increased dietary magnesium levels significantly upregulated the expressions of magnesium absorption‐related genes. At 2% calcium level groups, with increasing dietary magnesium levels, the expressions of intestinal calcium absorption‐related genes initially decreased then increased, while those of hepatopancreas calcium absorption‐related genes significantly increased. Furthermore, significant interactive effects between dietary calcium and magnesium levels were observed on WGR, carapace hardness, collagen fiber content, CHI activity, and molting hormone levels in hemolymph. In conclusion, the interaction between dietary calcium and magnesium levels significantly influenced the growth performance, exoskeleton development, and molting of *E. sinensis*. An increase in dietary calcium levels should be accompanied by an appropriate elevation in magnesium levels in formulated feeds for this species.

## 1. Introduction

Calcium is an essential mineral element for animals, involved in various metabolic processes and energy metabolism. It acts as a cofactor for numerous enzymes and serves as a key secondary messenger in signal transduction, playing an important role in the release of neurotransmitters and synaptic vesicle recycling [1–3]. Aquatic animals can acquire calcium ions either from the diet or through direct absorption via tissues such as gills and skin. The calcium requirements vary among species: fish such as Japanese seabass (*Lateolabrax japonicus*) [4], silver carp (*Hypophthalmichthys molitrix*) [5], and Indian catfish (*Heteropneustes fossilis*) [6] generally exhibit lower calcium demands than crustaceans. In contrast, crustaceans require higher dietary calcium than fish due to the demands of the molting process [7, 8]. Generally, more than 80% of the calcium in crustaceans is deposited in the exoskeleton [9]. The molting process involves shedding the old exoskeleton and forming a new one, resulting in a significant calcium loss. Calcium ions are critical for the mineralization of the new shell and are closely associated with its mechanical strength [10, 11]. Therefore, sufficient external calcium supply is essential to support post‐molt mineralization [12]. Inadequate calcium intake from water or feed may lead to unsuccessful molting, poor exoskeleton mineralization, reduced defensive capacity, and increased mortality [13, 14]. For instance, redclaw crayfish (*Cherax quadricarinatus*) fed a low‐calcium diet showed a significantly lower weight gain and survival rate (SR) compared to those fed a high‐calcium diet [14]. However, excessive dietary calcium can also adversely affect aquatic animals by inhibiting growth, impairing nutrient absorption and utilization [4], and causing skeletal deformities [6]. Thus, formulating feeds with appropriate calcium levels is essential for supporting the growth and molting of crustaceans.

Magnesium is an essential macromineral that acts as a cofactor for numerous enzymes, playing a fundamental role in the metabolism of proteins, carbohydrates, and lipids [15]. For instance, magnesium acts as a cofactor for key glycolytic enzymes such as hexokinase, phosphofructokinase, and pyruvate kinase, playing a critical role in regulating carbohydrate metabolism [16]. Additionally, it is involved in stress defense, immune responses, and bone metabolism [17, 18]. Aquatic animals primarily obtain magnesium from both water and the diet. Seawater contains high levels of magnesium, making dietary supplementation generally unnecessary for marine species [19]. In contrast, freshwater environments exhibit low magnesium concentrations, often insufficient to meet the metabolic demands of aquatic organisms [20]. Magnesium deficiency can lead to poor growth, high mortality, lethargy, and reduced bone strength [21–26]. In crustaceans, magnesium ions may regulate calcium absorption and the formation of new exoskeleton by modulating the activity of alkaline phosphatase [27, 28]. Moreover, magnesium contributes to exoskeleton formation by integrating with calcium carbonate as magnesium calcite, providing mechanical strength and structural support [29]. After molting, crustaceans require a substantial mineral intake from the environment for the synthesis of the new shell [30]. Therefore, dietary magnesium supplementation is crucial for freshwater crustaceans.

Calcium and magnesium ions share similar chemical properties, leading to competition for the common transport pathways. Specifically, magnesium can competitively inhibit calcium channels and binding sites, acting as a natural calcium antagonist [31]. For example, the transient receptor potential melastatin 7 channel is involved in the transport of both magnesium and calcium ions [32, 33]. Furthermore, magnesium serves as a cofactor for various ATPases, including Ca^2+^‐ATPase, thereby regulating calcium influx and efflux, thus influencing intracellular calcium homeostasis [34, 35]. Since calcium acts as a key second messenger, magnesium indirectly modulates a wide range of cellular functions [36]. To date, research on calcium–magnesium interactions in crustaceans has primarily focused on aquatic environments. For instance, the giant freshwater prawn (*Macrobrachium rosenbergii*) exhibited the highest SR when water calcium and magnesium concentrations reach 240 and 300 mg/kg, respectively [37]. In addition, elevated water calcium levels were shown to significantly reduce the whole‐body magnesium deposition in Chinese mitten crab (*Eriocheir sinensis*) [38]. However, studies on the interactions between dietary calcium and magnesium still remain absent.

Chinese mitten crab, also known as the river crab, is highly valued by consumers for its delicious taste and nutritional quality, making it one of the most important aquaculture species in China [39]. As a typical crustacean, its growth is intrinsically linked to the molting process [40]. In practical aquaculture, to support normal molting and healthy growth, farmers often incorporate calcium‐rich ingredients, such as fish meal and calcium phosphate, into formulated feeds. However, although magnesium plays a vital role in calcium homeostasis and exoskeleton development in crustaceans, the interaction between calcium and magnesium has received little attention. To date, the optimal dietary calcium‐to‐magnesium ratio for *E. sinensis* still remains unclear. Increasing dietary calcium without adjusting magnesium levels may lead to nutritional imbalance and reduced farming efficiency. Therefore, this study aims to investigate the interactive effects of dietary calcium and magnesium levels on the growth, tissue mineral deposition, exoskeleton development, and molting in *E. sinensis*. The findings will provide a crucial guidance for the reasonable adjustment of dietary calcium and magnesium contents in aquafeed.

## 2. Materials and Methods

### 2.1. Animal Ethics

All experimental protocols were approved by the Animal Care and Use Committee of the Nanjing Agricultural University (permit number: IACUC2019549). All animal experiments were performed in compliance with the Guidelines for the Care and Use of Laboratory Animals in China.

### 2.2. Experimental Design and Feed Formulation

Previous studies have reported that the optimal dietary calcium and magnesium requirements for *E. sinensis* are 1.5% [41] and 0.3%–0.45% [42], respectively. Based on these findings, a 2 × 4 factorial design was adopted in this study, comprising two targeted calcium levels (1% and 2%) and four targeted magnesium levels (0.15%, 0.3%, 0.45%, and 0.6%), resulting in eight experimental groups. Eight isonitrogenous and isolipidic diets were formulated accordingly, using Ca(H_2_PO_4_)_2_ and calcium chloride as calcium sources, and nanosized magnesium oxide as magnesium source. The composition and nutritional profiles of the experimental diets are presented in Table 1. All ingredients were ground using a grinder and passed through a 60‐mesh sieve. After weighing, they were thoroughly mixed in a stepwise manner. Approximately 30% distilled water was added to homogenize the mixture. The moist mixture was then pelleted using a twin‐screw extruder (Model F‐26, Guangdong, China) into cylindrical pellets with a diameter of 2.5 mm. The pellets were air‐dried at room temperature for 24 h and subsequently stored in sealed bags at –20°C until use.

**Table 1:** Formulation and proximate composition of the experimental diets (%).

Ingredients	Groups							
	1% Ca				2% Ca			
	0.15% Mg	0.3% Mg	0.45% Mg	0.6% Mg	0.15% Mg	0.3% Mg	0.45% Mg	0.6% Mg
Fish meal	5	5	5	5	5	5	5	5
Casein	32	32	32	32	32	32	32	32
Gelatin	9	9	9	9	9	9	9	9
Corn starch	25	25	25	25	25	25	25	25
Fish oil	3	3	3	3	3	3	3	3
Soybean oil	3	3	3	3	3	3	3	3
Lecithin	1	1	1	1	1	1	1	1
Cholesterol	0.5	0.5	0.5	0.5	0.5	0.5	0.5	0.5
Premix^a^	6	6	6	6	6	6	6	6
Choline chloride	0.5	0.5	0.5	0.5	0.5	0.5	0.5	0.5
Antioxidant	0.1	0.1	0.1	0.1	0.1	0.1	0.1	0.1
Cellulose	6.8	6.55	6.3	6.05	4.025	3.775	3.525	3.275
Carboxymethyl cellulose	2	2	2	2	2	2	2	2
Ca(H_2_PO_4_)_2_	5.85	5.85	5.85	5.85	5.85	5.85	5.85	5.85
Calcium chloride	0	0	0	0	2.775	2.775	2.775	2.775
MgO	0.25	0.5	0.75	1	0.25	0.5	0.75	1
Total	100	100	100	100	100	100	100	100
Proximate composition								
Moisture	8.19	8.05	8.04	8.58	8.40	8.66	8.70	8.25
Crude protein	39.08	38.83	38.82	38.99	39.12	39.05	38.93	38.85
Crude lipid	7.82	7.80	8.12	8.01	7.85	7.94	7.84	8.82
Crude ash	7.22	7.46	7.61	7.78	12.16	12.42	12.53	12.61
Calcium	1.01	1.01	1.02	1.01	1.89	1.96	1.94	2.04
Phosphorus	1.73	1.76	1.75	1.74	1.80	1.79	1.73	1.74
Magnesium	0.24	0.41	0.53	0.69	0.25	0.40	0.54	0.69

^a^Premix supplied the following minerals and vitamins (per kg of premix): CuSO_4_·5H_2_O 2 g, FeSO_4_·7H_2_O 25 g, ZnSO_4_·7H_2_O 22 g, MnSO_4_·4H_2_O 7 g, Na_2_SeO_3_ 0.04 g, KI 0.026 g, CoCl_2_·6H_2_O 0.1 g, Vitamin A, 900,000 IU; Vitamin D, 200,000 IU; Vitamin E, 4500 mg; Vitamin K_3_, 220 mg; Vitamin B_1_, 320 mg; Vitamin B_2_, 1090 mg; Vitamin B_5_, 2000 mg; Vitamin B_6_, 500 mg; Vitamin B_12_, 1.6 mg; Vitamin C, 10,000 mg; Pantothenate, 1000 mg; Folic acid, 165 mg; Biotin, 100 mg; Myoinositol 15,000 mg.

### 2.3. Experimental Animals and Rearing Management

The feeding trial was conducted at the Aquaculture Teaching and Research Base of Nanjing Agricultural University in Pukou District, Nanjing, Jiangsu Province, China. The experimental crabs were purchased from a local farm. Before the trial began, the crabs were acclimatized for 1 week by being fed a commercial diet (Jiangsu Haipurui Feed Co., Ltd, China). A total of 240 healthy crabs with intact appendages and an initial body weight of 40.46 ± 0.47 g were randomly distributed into 8 groups, each fed one of the experimental diets. Each group consisted of three replicates with 10 crabs per replicate. The crabs were reared in 24 outdoor concrete tanks (1.0 × 1.0 × 0.8 m; L:W:H). Each tank was provided with 12 white PVC tubes (length: 20 cm; diameter: 15 cm) as shelters. The trial lasted for 10 weeks. The crabs were fed daily at 20:00 at a rate of 4% of their body weights. Every week, three crabs were randomly selected from each replicate and weighed to determine the feeding amount. Uneaten feed was removed after 2 h. The number of molts and mortalities was recorded daily. One‐third of the water was exchanged each day. Water temperature was maintained at 28°C–30°C, pH varies between 7.3 and 7.5, dissolved oxygen was set above 5 mg/L, total ammonia nitrogen was kept below 0.05 mg/L, calcium and magnesium concentrations were ~35 and 10 mg/L, respectively.

### 2.4. Sample Collection

At the end of the feeding trial, all experimental crabs were fasted for 24 h to clear their gastrointestinal tracts. Crabs from each replicate were then counted, anesthetized on ice, and weighed to calculate growth performance indicators. Subsequently, four crabs (in the intermolt stage) per replicate were randomly selected. Hemolymph was collected from the base of the fourth walking leg using a 1 mL sterile syringe and was immediately mixed with an anticoagulant solution (13.2 g/L trisodium citrate, 4.8 g/L citric acid, 14.7 g/L glucose, in 1 L distilled water) at a 1:1 ratio. The mixture was centrifuged at 4°C and 4500 rpm for 10 min. The resulting supernatant was collected and stored at −20°C for subsequent analysis. The crabs were then promptly dissected with the hepatopancreas, intestine, muscle, exoskeleton and the inner epidermis of the carapace excised. The hepatopancreas was then weighed. A 1–2 cm section of the carapace from the branchial region was clipped and fixed in 4% paraformaldehyde for histological analysis. The samples were immediately placed in cryovials and stored in a −80°C freezer for later use. One intact crab from each replicate was reserved and stored at −20°C for the assessment of carapace hardness and the whole body calcium and magnesium deposition.

### 2.5. Proximate Composition Analysis

The proximate composition of the experimental diet was measured following the standard methods [43]. After drying the sample at 105°C to a constant weight, the moisture was calculated. Crude protein was determined using the Auto Kjeldahl System (Kjeltec, Foss, Sweden). Crude ash was examined by burning the samples for 4 h at 550°C. Crude lipid was measured using the Soxhlet extraction method (Soxtec, Tecator, Sweden).

### 2.6. Measurement of Carapace Hardness

Carapace hardness was measured according to a previously described method [38] and made several modifications. The cardiac region of the carapace was selected as the test area. Measurements were performed using a texture analyzer (TA.XT Plus, Stable Micro Systems, Surrey, UK) equipped with a 50 mm aluminum compression plate. The settings were as follows: a pre‐test speed of 1 mm/s, a trigger force of 1 N, and a deformation level of 20%. All procedures were automatically controlled and recorded by the instrument. Three crabs were randomly selected from each group for the measurement.

### 2.7. Histological Analysis of the Carapace

An individual within each treatment was randomly selected for the histological analysis of the carapace. Sections of the carapace from the gill region were decalcified in a 10% hydrochloric acid‐formalin solution, dehydrated through a graded ethanol series, cleared in xylene, and embedded in paraffin. Transverse sections of 6 μm thickness were prepared and stained using Masson’s trichrome method to visualize collagen fibers. The relative collagen area was quantified using the ImageJ software.

### 2.8. Determination of Diet and Tissue Mineral Deposition

Diet calcium, phosphorus and magnesium contents and tissues (hepatopancreas, intestine, muscle, exoskeleton, and the whole body) calcium and magnesium contents were all determined by inductively coupled plasma optical emission spectrometry (ICP‐OES). Sample preparation was performed via microwave digestion. Microwave digestion vessels were soaked in 10% nitric acid for 12 h, then thoroughly cleaned using an ultrasonic cleaner (Model SK5210HP, Kedao Ultrasonic Instrument Co., Ltd, Shanghai). After drying in an oven at 50°C, ~0.2 g of sample was weighed into each vessel, with two blank vessels prepared as controls. Ten milliliters of concentrated nitric acid (analytical grade) were added to each vessel. Digestion was carried out using a microwave digestion system (CEM Mars6, USA) with the following program: ramping to 120°C in 5 min and holding for 5 min; ramping to 150°C in 5 min and holding for 10 min; ramping to 190°C in 5 min and holding for 30 min. After the temperature decreased below 60°C, the vessels were carefully vented. The digests were then evaporated at 150°C for 2 h on a heating block (Boton Biotechnology Co., Ltd, Shanghai) to remove residual acid. After cooling to room temperature, the samples were diluted to 50 mL with ultrapure water and mixed thoroughly for ICP‐OES analysis (Thermo iCAP 7000, USA). Calcium and magnesium concentrations in hemolymph were measured using commercial assay kits (Calcium Assay Kit, Cat. No. C004‐2‐1; Magnesium Assay Kit, Cat. No. C005‐1‐1; Nanjing Jiancheng Bioengineering Institute, China) strictly following the manufacturer’s instructions.

### 2.9. Measurement of Molting‐Related Hormones Concentrations

The concentrations of ecdysone and methyl farnesoate (MF) in the hemolymph were determined as described by Xiong et al. [44] and He et al. [45], respectively.

### 2.10. Measurement of Epidermal Enzyme Activities

The activities of chitinase (CHI) and β‐N‐acetylglucosaminidase (β‐NAG) in the epidermis were measured according to a previous study [44].

### 2.11. Measurement of Gene Expression Levels

#### 2.11.1. Total RNA Extraction

Approximately 0.1 g of hepatopancreas and intestine was homogenized with total RNA extracted using a commercial RNA extraction kit (Accurate Biology, Hunan, China). The concentration and purity of the extracted RNA were assessed using a microspectrophotometer (NanoDrop Technologies, Wilmington, USA) with RNA quality evaluated based on the absorbance ratio at 260 and 280 nm.

#### 2.11.2. Reverse Transcription

Complementary DNA (cDNA) was synthesized from 1 μg of total RNA using a SYBR Prime Script RT reagent kit (Accurate Biology, Hunan, China) according to the manufacturer’s instructions. The resulting cDNA was stored at –20°C for subsequent quantitative real‐time (qPCR) analysis.

#### 2.11.3. qPCR

The expression levels of genes related to molting and calcium/magnesium absorption were quantified using a SYBR Green II fluorescence kit (Accurate Biology, Hunan, China) on a QuantStudio7 Flex Real‐Time PCR System (Thermo Fisher, USA). Each 20 μL reaction mixture contained 10 μL of 2 × SYBR Green real‐time PCR master mix, 0.4 μL of each of forward and reverse primers, 2 μL of cDNA template, and 7.2 μL of DEPC‐treated water. The amplification protocols consisted of an initial denaturation at 95°C for 30 s, followed by 40 cycles of denaturation at 95°C for 10 s, annealing at 60°C for 28 s, and extension at 72°C for 30 s. A melt curve analysis was performed as follows: 95°C for 15 s, 60°C for 1 min, and then 95°C for 15 s. All primer sequences used are listed in Table 2, and were designed and synthesized by Sangon Biotech (Shanghai, China). The ubiquitin/ribosomal S27 fusion protein gene was used as an internal reference [46], and relative gene expression levels were calculated using the 2^−ΔΔCt^ method [49].

**Table 2:** Primer pair sequences for real‐time PCR analysis.

Gene names	Forward sequences (5′–3′)	Reverse sequences (3′–5′)	Accession numbers/References
*s27*	GGTCGATGACAATGGCAAGA	CCACAGTACTGGCGGTCAAA	[46]
*chh*	GCTACAGCAACCTCGTCTTCCG	TTCTTCCTGCCAACCACCC	JX485644.1
*ecr*	CTCCCGGGTGCCATATTACC	TGCTACACGGCACATTCACT	KF469223.1
*rxr*	ACCCTGTGCTAACCCTCTGA	TGCTCACCACATCCTGCTTT	MK604180.1
*mih*	TTTAGCTCCGTTCACGCCTT	TGGAGAACCCAGGAAAGCAC	DQ341280.1
*crt*	GATCGACAACCCCGAGTACACC	TGCTTCGCCTCCTCAATATCGTC	KU519400.1
*cnx*	TGGAGCCCTTCAAAATGAGACCC	CCTCAGCCGTATTGTCAGTCAC	KU519399.1
*nipa2*	CCGCTGCTGATTCCTACTGG	GCGACTGTGCTGAACTGTTATT	XM050857575.1
*cpcbm*	CTGTTGCCTCA TCCCGAAAA	ATTGTACTCCCAGTTGCATGTCAC	[47]
*chi*	GAGCCCTACGTCTACAGCATCAC	GGTCTCAACACTCCAAACCATCA	KJ513466.1
*chs*	AGAACCCTCAGGAGCAGAGAA	GCCTCGACGAAGGTGATGTT	XM050862404.1
*bmp2*	ATCGTAGTCCCAGCAGTCCT	CTGGTGGTACGCAACAAAGC	[48]

Abbreviations: *bmp2*, bone morphogenetic proteins 2; *chi*, chitinase; *chs*, chitin synthase; *chh*, crustacean hyperglycemic hormone; *cnx*, calnexin; *cpcbm*, cuticle protein cbm; *crt*, calreticulin; *ecr*, ecdysone receptor; *mih*, molt‐inhibiting hormone; *nipa2*, non‐imprinted in Prader–Willi/Angelman syndrome region protein 2; *rxr*, retinoid X receptor; *s27*, ubiquitin/ribosomal S27 fusion protein.

### 2.12. Statistical Analysis

All data were analyzed using two‐way analysis of variance (Two‐way ANOVA) in SPSS 27.0. If a significant interaction (*p* < 0.05) was detected, one‐way ANOVA followed by Tukey’s multiple comparison test was applied to compare different magnesium levels within the same calcium level. For comparisons between the two calcium levels at the same magnesium level, an independent samples *t*‐test was used. All data are presented as mean ± standard error of the mean (SEM).

## 3. Results

### 3.1. Growth Performance and Feed Utilization

As shown in Table 3, no significant difference was observed in final body weight (FBW), SR, feed intake (FI), and feed conversion ratio (FCR) among the experimental groups (*p* > 0.05). High calcium level significantly decreased the hepatosomatic index (HSI), molting ratio (MR) and carapace hardness (*p* < 0.05). However, for weight gain rate (WGR) and specific growth rate (SGR), the significant decrease was only observed in the 0.3% magnesium level group. Moreover, with increasing magnesium levels, the carapace hardness initially increased, followed by a subsequent decrease (*p* < 0.05). Although the difference was not statistically significant, a trend toward an increase in WGR was observed. Significant interactive effects between dietary calcium and magnesium levels were detected on WGR, SGR, MR, and carapace hardness (*p* < 0.05). In 1% calcium level, the maximum values of WGR and SGR were observed in 0.3% magnesium group, while in 2% calcium level, the maximum values of WGR and SGR were observed in 0.6% magnesium group.

**Table 3:** Effects of different dietary calcium and magnesium levels on the growth performance and feed utilization of *Eriocheir sinensis*.

	CL	ML				Two‐way ANOVA (*p* value)		
		0.15%	0.3%	0.45%	0.6%	CL	ML	CL × ML
IBW (g)	1%	40.35 ± 0.42	40.35 ± 0.15	40.67 ± 0.5	40.55 ± 0.09	0.836	0.830	0.863
	2%	40.38 ± 0.17	40.54 ± 0.32	40.6 ± 0.19	40.21 ± 0.44			
FBW (g)	1%	86.22 ± 2.88	88.7 ± 2.47	83.06 ± 3.5	83.93 ± 2.23	0.294	0.626	0.131
	2%	82.69 ± 2.92	79.99 ± 2.77	82.35 ± 2.29	88.65 ± 2.15			
WGR (%)	1%	113.56 ± 4.88	119.81 ± 5.8	104.18 ± 3.73	107 ± 5.92	0.196	0.279	0.021
	2%	104.91 ± 4.68	97.25 ± 5.44 ^∗^	102.36 ± 5.42	120.47 ± 4.76			
SGR (%/d)	1%	1.08 ± 0.03	1.12 ± 0.04	1.02 ± 0.03	1.04 ± 0.04	0.188	0.287	0.022
	2%	1.02 ± 0.03	0.97 ± 0.04 ^∗^	1.01 ± 0.04	1.13 ± 0.03			
SR (%)	1%	63.33 ± 8.82	50 ± 0	70 ± 10	66.67 ± 12.02	0.664	0.245	0.936
	2%	60 ± 5.77	53.33 ± 3.33	66.67 ± 12.02	60 ± 0			
HSI (%)	1%	7.05 ± 0.23	7.74 ± 0.33	6.65 ± 0.75	7.33 ± 0.61	0.011	0.100	0.739
	2%	5.28 ± 0.18	6.84 ± 0.58	5.6 ± 0.24	6.7 ± 0.88			
FI (g)	1%	102.58 ± 16.48	128.19 ± 2.92	95.14 ± 13.11	105.82 ± 13.67	0.723	0.102	0.942
	2%	111.24 ± 8.04	123.51 ± 7.34	97.65 ± 14.55	110.63 ± 2.97			
FCR	1%	2.22 ± 0.27	2.67 ± 0.19	2.26 ± 0.34	2.45 ± 0.35	0.285	0.121	0.74
	2%	2.65 ± 0.28	3.14 ± 0.17	2.33 ± 0.27	2.29 ± 0.1			
MR	1%	1.35 ± 0.01	1.42 ± 0.04	1.26 ± 0.06	1.25 ± 0.05	0.038	0.223	0.015
	2%	1.29 ± 0.02	1.18 ± 0.03 ^∗∗^	1.19 ± 0.06	1.33 ± 0.04			
Carapace hardness (N)	1%	4.67 ± 0.11^a^	3.51 ± 0.03^b^	5.18 ± 0.09^a^	3.07 ± 0.18^b^	<0.001	<0.001	<0.001
	2%	4 ± 0.1A ^∗^	3.53 ± 0.2^A^	2.56 ± 0.13^B^^∗∗∗^	3.54 ± 0.33^A^			

*Note:* Weight gain rate (WGR, %) = 100 × (FBW − IBW)/IBW. Specific growth rate (SGR, %/day) = 100 × (ln FBW − ln IBW)/days of experiment. Survival rate (SR, %) = 100 × final numbers of crabs/initial numbers of crabs. Hepatosomatic index (HSI, %) = 100 × hepatopancreas weight/corresponding crab body weight. Feed intake (FI, g/crab) = total feed consumption per replicate/number of crabs per replicate. Feed conversion ratio (FCR) = total feed consumption per replicate/total weight gain of surviving and dead crabs per replicate. Molting ratio (MR) = total number of molts/[(initial number of crabs + final number of crabs)/2]. Different letters (lowercase for 1% calcium level and uppercase for 2% calcium level) indicate significant differences (*p* < 0.05) among different magnesium levels at the same calcium level.

Abbreviations: CL, calcium level; FBW, final body weight; IBW, initial body weight; ML, represents the magnesium level; ns, no significant difference.

^∗^indicates significant differences (*p* < 0.05) between different calcium levels at the same magnesium level.

^∗^ *p* < 0.05,  ^∗∗^ *p* < 0.01,  ^∗∗∗^ *p* < 0.001.

### 3.2. Tissue Mineral Deposition

#### 3.2.1. Calcium Deposition

As presented in Table 4, hemolymph calcium content showed no statistical difference (*p* > 0.05). A dietary calcium level of 2% significantly reduced the calcium content in the intestine and muscle, but significantly increased the whole‐body and exoskeleton calcium content (*p* < 0.05). Elevated magnesium levels significantly increased the calcium content in the hepatopancreas, muscle, and exoskeleton (*p* < 0.05). Hepatopancreas calcium content showed an increasing trend with rising magnesium levels, while calcium content in muscle and the exoskeleton first decreased then increased. Significant interactive effects between dietary calcium and magnesium levels were observed on calcium content in the hepatopancreas, muscle, and exoskeleton (*p* < 0.05). The maximum calcium deposition in the exoskeleton was observed in the 2% calcium and 0.6% magnesium group.

**Table 4:** Effects of different dietary calcium and magnesium levels on calcium deposition (wet‐weight basis) in various tissues of *Eriocheir sinensis*.

	CL	ML				Two‐way ANOVA (*p* value)		
		0.15%	0.3%	0.45%	0.6%	CL	ML	CL × ML
Hemolymph (mmol/L)	1%	8.52 ± 0.19	7.79 ± 0.2	8.64 ± 0.26	7.4 ± 0.86	0.818	0.389	0.339
	2%	7.33 ± 1.05	8.58 ± 0.46	8.79 ± 0.43	8.05 ± 0.63			
Hepatopancreas (mg/kg)	1%	0.6 ± 0.03^b^	1.04 ± 0.08^ab^	0.98 ± 0.07^b^	3.14 ± 1^a^	0.013	0.012	0.006
	2%	0.6 ± 0.09	0.76 ± 0.08 ^∗^	0.96 ± 0.28	0.63 ± 0.04			
Intestine (mg/kg)	1%	0.69 ± 0.07	0.84 ± 0.16	1.07 ± 0.09	0.94 ± 0.04	<0.001	0.002	0.678
	2%	0.45 ± 0.04	0.56 ± 0.05	0.85 ± 0.13	0.53 ± 0.01			
Muscle (mg/kg)	1%	3.99 ± 0.65^a^	0.96 ± 0.35^b^	3.13 ± 0.57^ab^	2.75 ± 0.64^ab^	<0.001	0.004	0.015
	2%	1.09 ± 0.27^B^^∗∗^	1.13 ± 0.18^B^	2.19 ± 0.31^A^	1.37 ± 0.21^AB^			
Whole body (mg/kg)	1%	212.07 ± 8.6	155.44 ± 23.54	97.55 ± 25.84	197.87 ± 4.27	<0.001	<0.001	0.200
	2%	231.07 ± 9.52	225.09 ± 8.91	175.27 ± 16.89	273.98 ± 10.2			
Exoskeleton (mg/kg)	1%	282.31 ± 5.92^a^	265.16 ± 4.71^ab^	249.43 ± 11.63^b^	282.66 ± 6.72^a^	0.601	<0.001	<0.001
	2%	217 ± 3.69^C^^∗∗∗^	280.67 ± 5.45^B^	236.13 ± 6^BC^	360.95 ± 22.44^A^^∗^			

*Note:* Different letters (lowercase for 1% calcium level and uppercase for 2% calcium level) indicate significant differences (*p* < 0.05) among different magnesium levels at the same calcium level.

Abbreviations: CL, calcium level; ML, magnesium level; ns, no significant difference.

^∗^indicates significant differences (*p* < 0.05) between different calcium levels at the same magnesium level.

^∗^ *p* < 0.05,  ^∗∗^ *p* < 0.01,  ^∗∗∗^ *p* < 0.001.

#### 3.2.2. Magnesium Deposition

As shown in Table 5, hemolymph magnesium content showed no statistical difference (*p* > 0.05). The high calcium diet significantly decreased magnesium content in the hepatopancreas and muscle, but significantly increased whole‐body magnesium content (*p* < 0.05). Dietary magnesium level significantly influenced magnesium content in the hemolymph, hepatopancreas, whole body, and exoskeleton (*p* < 0.05). Increasing magnesium levels significantly raised hepatopancreas magnesium content (*p* < 0.05). A significant interactive effect between dietary calcium and magnesium levels was detected on hepatopancreas magnesium content (*p* < 0.01), which peaked in the 1% calcium and 0.6% magnesium group.

**Table 5:** Effects of different dietary calcium and magnesium levels on magnesium deposition (wet‐weight basis) in various tissues of *Eriocheir sinensis*.

	CL	ML				Two‐way ANOVA (*p* value)		
		0.15%	0.3%	0.45%	0.6%	CL	ML	CL × ML
Hemolymph (mmol/L)	1%	2.41 ± 0.18	5.12 ± 0.28	3.44 ± 0.44	3.19 ± 0.36	0.584	<0.001	0.256
	2%	2.93 ± 0.34	5.43 ± 0.72	4.32 ± 0.76	2.22 ± 0.4			
Hepatopancreas (mg/kg)	1%	0.23 ± 0.01^b^	0.29 ± 0.01^b^	0.29 ± 0^b^	0.49 ± 0.07^a^	0.007	<0.001	0.001
	2%	0.25 ± 0^B^	0.25 ± 0.01^B^^∗^	0.3 ± 0.02^A^	0.3 ± 0.01^A^^∗^			
Intestine (mg/kg)	1%	0.24 ± 0.01	0.26 ± 0.01	0.24 ± 0.02	0.23 ± 0.05	0.307	0.628	0.230
	2%	0.19 ± 0.02	0.22 ± 0.02	0.24 ± 0.01	0.26 ± 0.03			
Muscle (mg/kg)	1%	0.35 ± 0.05	0.3 ± 0.02	0.33 ± 0.04	0.3 ± 0.04	0.002	0.529	0.671
	2%	0.26 ± 0.01	0.27 ± 0.02	0.24 ± 0.01	0.23 ± 0.02			
Whole body (mg/kg)	1%	4.61 ± 0.39	4.58 ± 0.64	2.92 ± 0.67	5.46 ± 0.41	<0.001	<0.001	0.964
	2%	6.62 ± 0.19	6.33 ± 0.36	5.15 ± 0.49	7.65 ± 0.7			
Exoskeleton (mg/kg)	1%	4.64 ± 0.74	6.28 ± 0.44	5.39 ± 0.75	7.78 ± 1.35	0.162	0.026	0.451
	2%	4.45 ± 0.26	6.36 ± 0.66	4.6 ± 0.84	5.58 ± 0.5			

*Note:* Different letters (lowercase for 1% calcium level and uppercase for 2% calcium level) indicate significant differences (*p* < 0.05) among different magnesium levels at the same calcium level.

Abbreviations: CL, calcium level; ML, magnesium level; ns, no significant difference.

^∗^indicates significant differences (*p* < 0.05) between different calcium levels at the same magnesium level.

^∗^ *p* < 0.05.

### 3.3. The Expressions of Genes Related to Calcium and Magnesium Absorption

#### 3.3.1. The Expressions of Genes in the Hepatopancreas

As shown in Figure 1, high calcium level significantly downregulated the expressions of genes encoding calreticulin (*crt*), calnexin (*cnx*), and non‐imprinted in Prader–Willi/Angelman syndrome region protein 2 (*nipa2*) in the hepatopancreas (*p*  < 0.05). Elevated magnesium levels significantly upregulated the expressions of *crt*, *cnx*, and *nipa2* (*p*  < 0.05). Significant interactive effects were observed on the expressions of *crt*, *cnx*, and *nipa2* in the hepatopancreas (*p*  < 0.05). The highest expression of crt was observed in the 2% calcium and 0.6% magnesium group, while the highest expression of *nipa2* was observed in the 1% calcium and 0.6% magnesium group.

Figure 1Effects of different dietary calcium and magnesium levels on the expression levels of genes related to calcium and magnesium absorption in the hepatopancreas of *Eriocheir sinensis*. Different letters (lowercase for 1% calcium level and uppercase for 2% calcium level) indicate significant differences (*p* < 0.05) among different magnesium levels at the same calcium level.  ^∗^ indicates significant differences (*p* < 0.05) between different calcium levels at the same magnesium level.  ^∗^ *p* < 0.05,  ^∗∗^ *p* < 0.01,  ^∗∗∗^ *p* < 0.001, ns: no significant difference. Data were normalized to the 1% Ca and 0.15% Mg group. (A) *c*rt, Calreticulin; (B) *cnx*, Calnexin; (C) *nipa2*, non‐imprinted in Prader‐Willi/Angelman syndrome region protein 2. CL represents the calcium level, ML represents the magnesium level.

**Figure:**
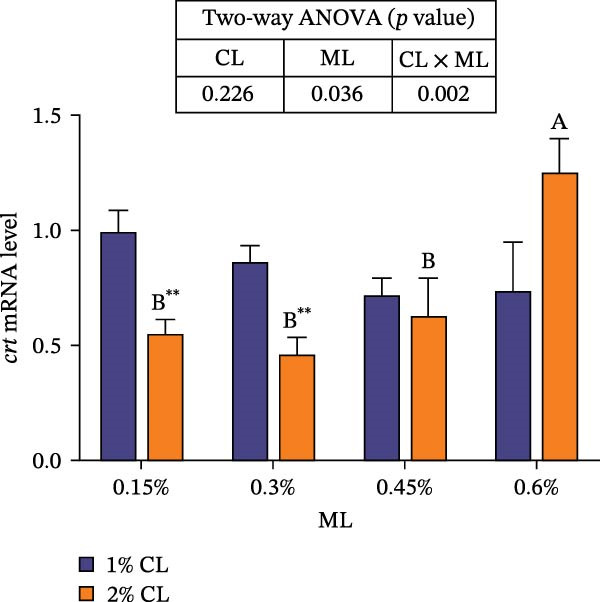
(A)

**Figure:**
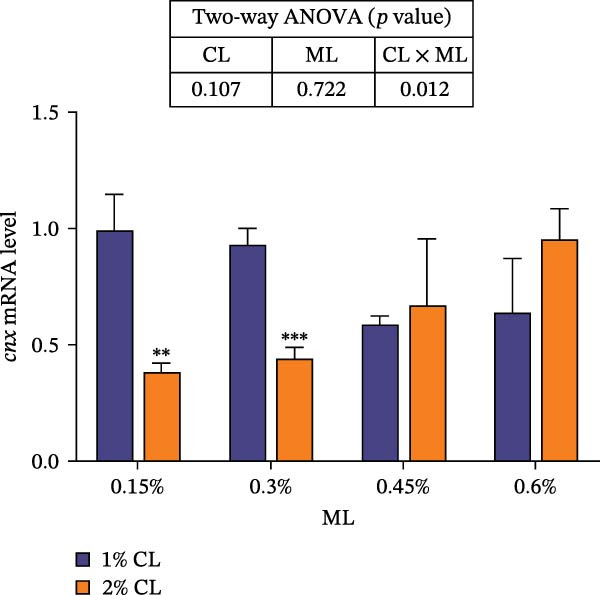
(B)

**Figure:**
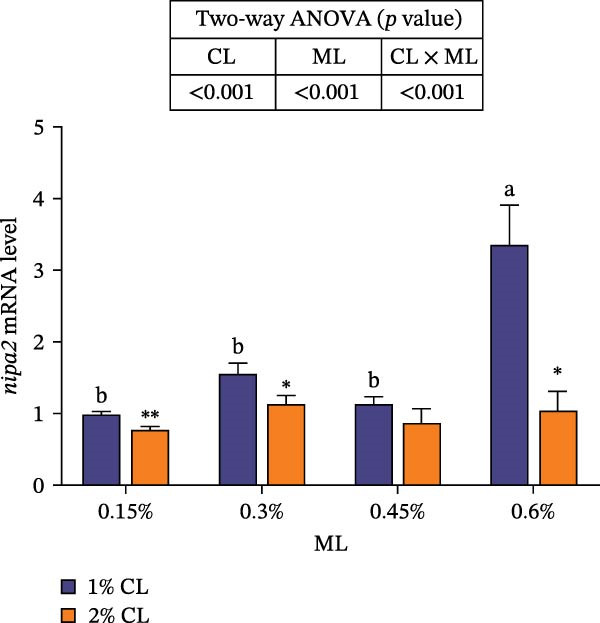
(C)

#### 3.3.2. The Expressions of Genes in the Intestine

As illustrated in Figure 2, high calcium level significantly upregulated the expressions of *cnx* and *nipa2 i*n the intestine (*p* < 0.05). The expressions of *crt* and *cnx* initially increased, then decreased and finally increased again with increasing magnesium levels, while the expression of *nipa2* was significantly reduced (p  < 0.05) in the 2% calcium level groups. Significant interactive effects were detected on the intestinal expressions of *crt*, *cnx*, and *nipa2* (*p* < 0.05). The expressions of *crt* and *cnx* achieved the highest value in the 2% calcium and 0.6% magnesium group.

Figure 2Effects of different dietary calcium and magnesium levels on the expression levels of genes related to calcium and magnesium absorption in the intestine of *Eriocheir sinensis*. Different letters (lowercase for 1% calcium level and uppercase for 2% calcium level) indicate significant differences (*p* < 0.05) among different magnesium levels at the same calcium level.  ^∗^ indicates significant differences (*p* < 0.05) between different calcium levels at the same magnesium level.  ^∗^ *p* < 0.05,  ^∗∗^ *p* < 0.01,  ^∗∗∗^ *p* < 0.001, ns: no significant difference. Data were normalized to the 1% Ca and 0.15% Mg group. (A) *c*rt, Calreticulin; (B) *cnx*, Calnexin; (C) *nipa2*, non‐imprinted in Prader‐Willi/Angelman syndrome region protein 2. CL represents the calcium level, ML represents the magnesium level.

**Figure:**
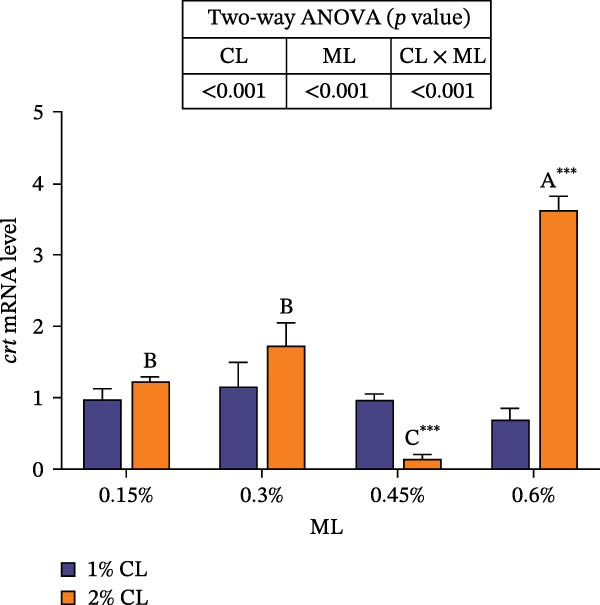
(A)

**Figure:**
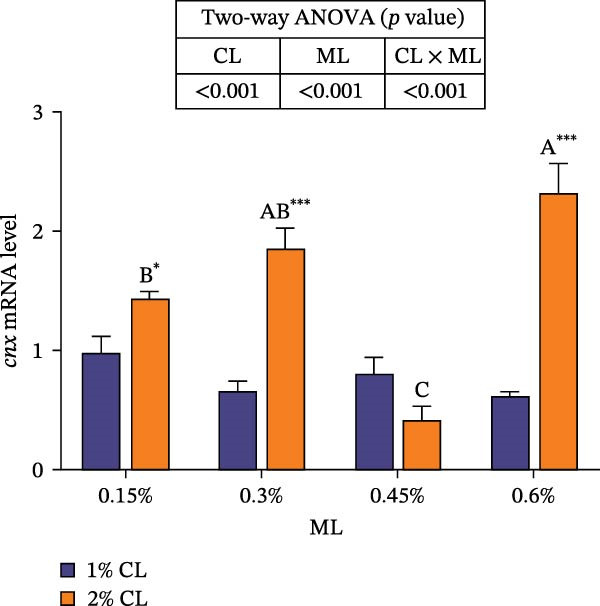
(B)

**Figure:**
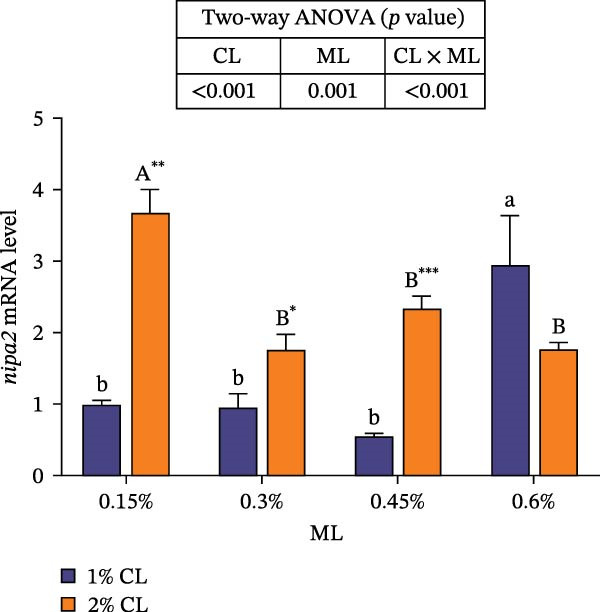
(C)

### 3.4. Carapace Structure and Relative Collagen Fiber Area

As shown in Figure 3, the carapace structure of intermolt *E. sinensis* was similar to that of other crustaceans, consisting of four distinct layers from exterior to interior: the epicuticle, exocuticle, endocuticle, and membranous layer. Masson’s trichrome staining revealed that the epicuticle is stained red with minimal collagen fibers, while the exocuticle and endocuticle is stained blue with abundant collagen fibers. The membranous layer appeared light blue with sparse collagen content. Quantitative analysis indicated that high calcium level significantly increased the relative collagen area in the carapace (*p* < 0.05). A significant interactive effect between dietary calcium and magnesium levels was observed on the relative collagen area of the carapace (*p* < 0.05). At 1% calcium level groups, the relative collagen area showed a decreasing trend with increasing magnesium levels, while at 2% calcium level groups, increasing the magnesium level elevated the relative collagen area. The maximum value was observed in the 2% calcium and 0.6% magnesium group.

Figure 3Location of collagen fibers (A–H) and relative collagen area (I) of the carapace of *Eriocheir sinensis*. Note: A: 1% Ca and 0.15% Mg, B: 1% Ca and 0.30% Mg, C: 1% Ca and 0.45% Mg, D: 1% Ca and 0.60% Mg, E: 2% Ca and 0.15% Mg, F: 2% Ca and 0.30% Mg, G: 2% Ca and 0.45% Mg, H: 2% Ca and 0.60% Mg. Ep, epicuticle; Ex, exocuticle; Ed, endEocuticle; MI, membranous layer. Scale bar: 50 μm. Relative collagen area = (collagen fiber area/total carapace cross‐sectional area) × 100%. CL represents the calcium level, ML represents the magnesium level. Different letters (lowercase for 1% calcium level and uppercase for 2% calcium level) indicate significant differences (*p* < 0.05) among different magnesium levels at the same calcium level.  ^∗^ indicates significant differences (*p* < 0.05) between different calcium levels at the same magnesium level.  ^∗^ *p* < 0.05, ns: no significant difference.

**Figure:**
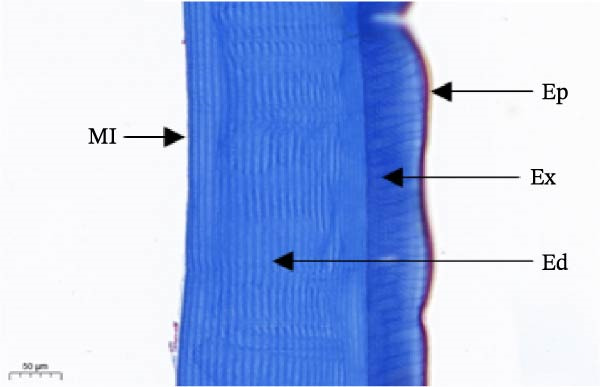
(A)

**Figure:**
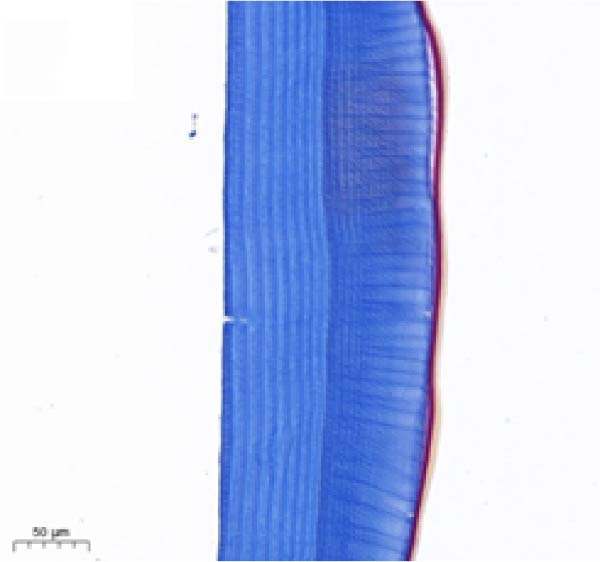
(B)

**Figure:**
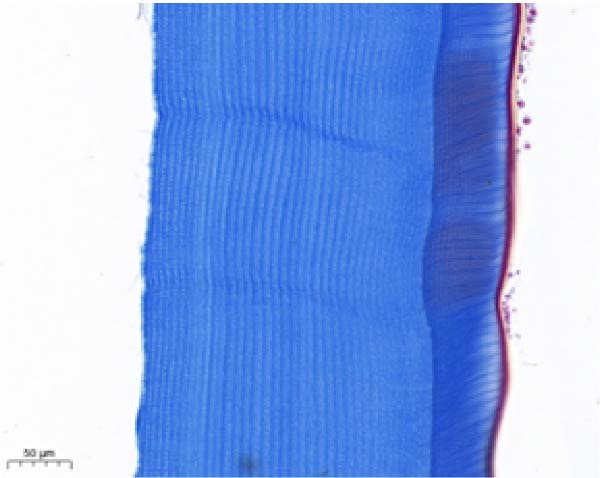
(C)

**Figure:**
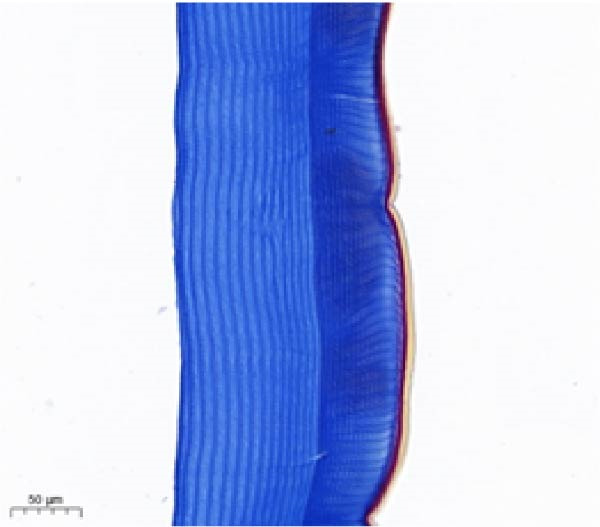
(D)

**Figure:**
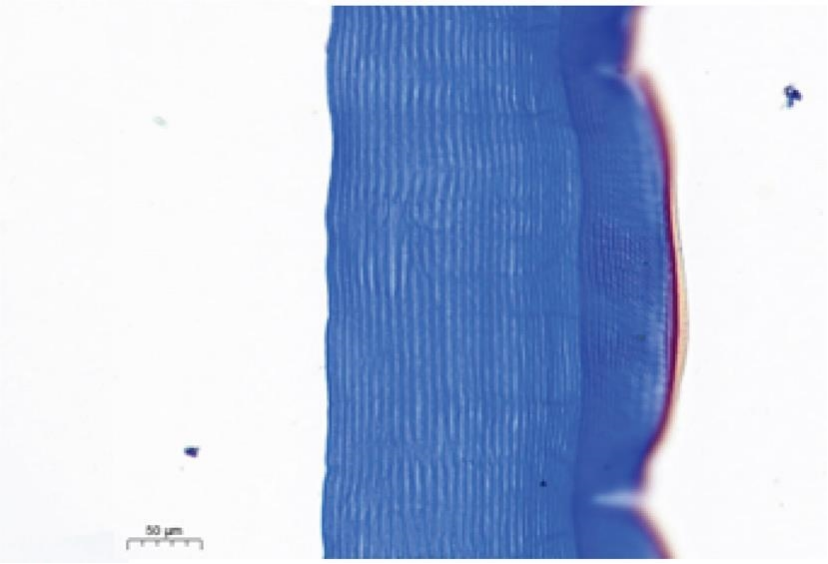
(E)

**Figure:**
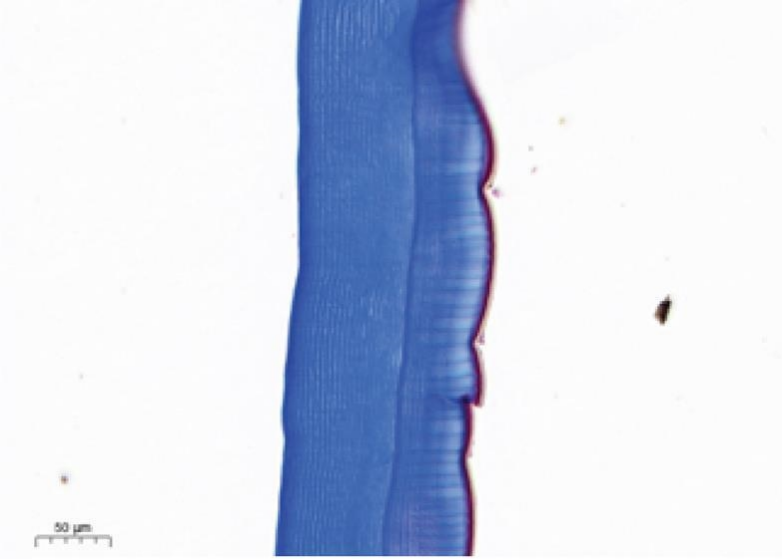
(F)

**Figure:**
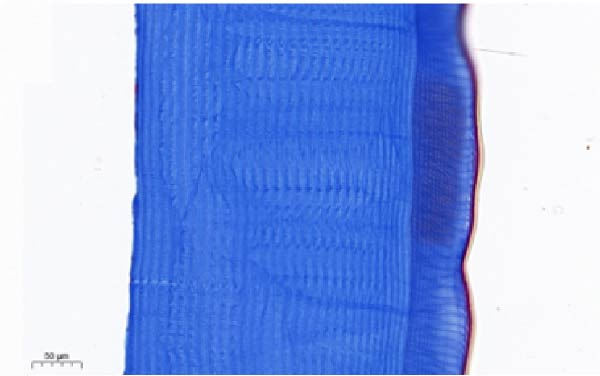
(G)

**Figure:**
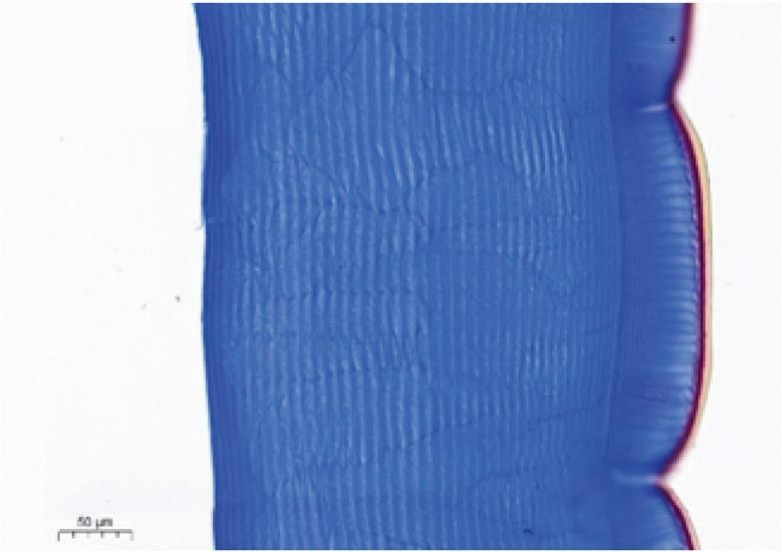
(H)

**Figure:**
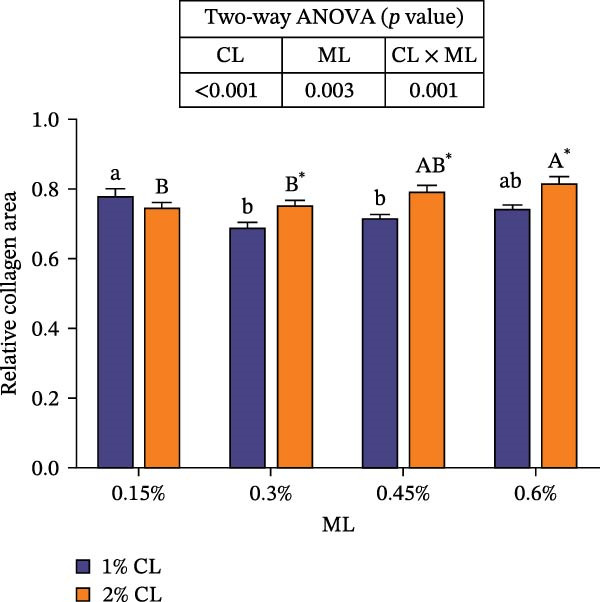
(I)

### 3.5. The Expressions of Genes Related to Exoskeleton Development

As presented in Figure 4, high calcium significantly upregulated the expressions of chitinase (*chi*), while downregulated the expressions of chitin synthase (*chs*) and bone morphogenetic proteins 2 (*bmp2*) (*p* < 0.05) at 0.3% magnesium level. The effects of calcium on cuticle protein cbm (*cpcbm*) expression were dependent on magnesium levels. At 0.15%–0.3% magnesium level, high calcium significantly upregulated the expression of *cpcbm*, while an opposite result was observed at 0.6% magnesium level (*p* < 0.05). With increasing magnesium levels, the expression of *chi* initially decreased then increased, while the expression of *chs* initially increased then decreased (*p* < 0.05). Significant interactive effects between calcium and magnesium levels were detected on the expressions of *chi*, *chs*, and *cpcbm* (*p* < 0.05). Elevated magnesium levels significantly increased the expression of *cpcbm* in the 1% calcium groups, while decreased it in the 2% calcium groups. The maximum value was observed in the 1% calcium and 0.6% magnesium group.

Figure 4Effects of different dietary calcium and magnesium levels on the expression levels of genes related to exoskeleton development in the hepatopancreas of *Eriocheir sinensis*. Different letters (lowercase for 1% calcium level and uppercase for 2% calcium level) indicate significant differences (*p* < 0.05) among different magnesium levels at the same calcium level.  ^∗^ indicates significant differences (*p* < 0.05) between different calcium levels at the same magnesium level.  ^∗∗^ *p* < 0.01,  ^∗∗∗^ *p* < 0.001, ns: no significant difference. Data were normalized to the 1% Ca and 0.15% Mg group. (A) *c*hi, Chitinase; (B) *c*hs, Chitin synthase; (C) *c*pcbm, Cuticle protein cbm; (D) *b*mp2, Bone morphogenetic proteins 2. CL represents the calcium level, ML represents the magnesium level.

**Figure:**
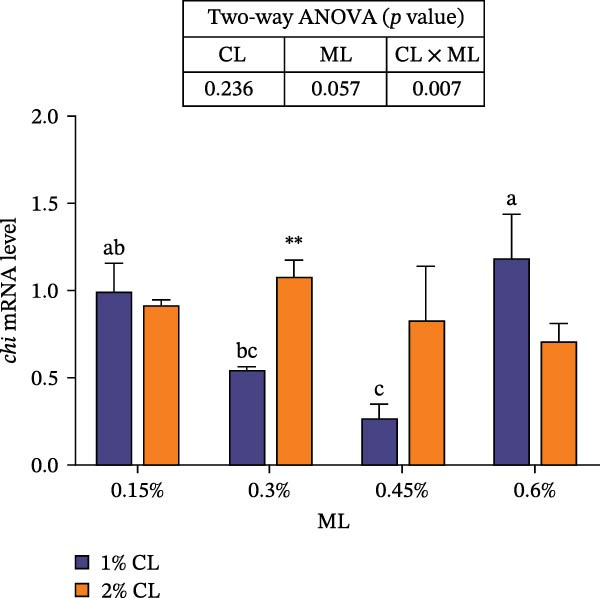
(A)

**Figure:**
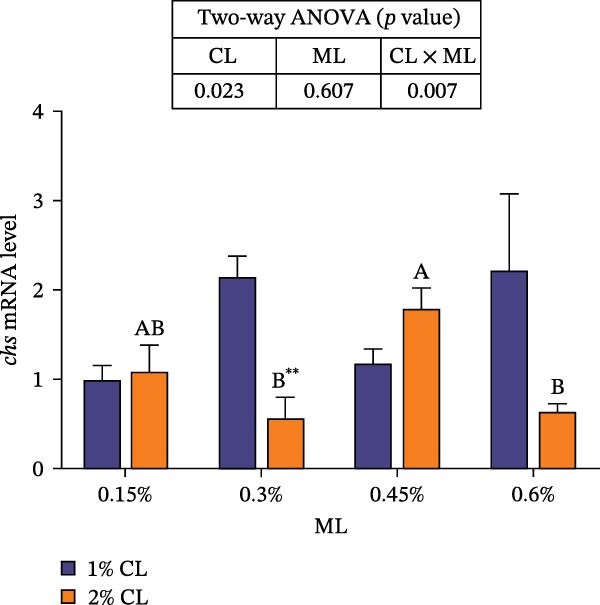
(B)

**Figure:**
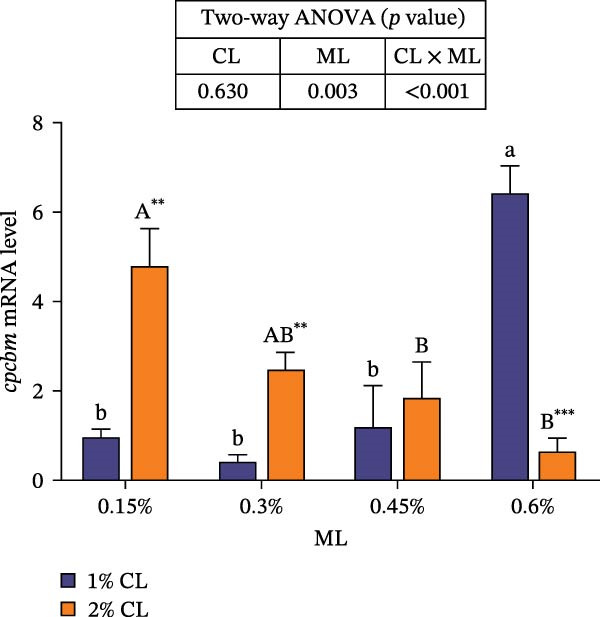
(C)

**Figure:**
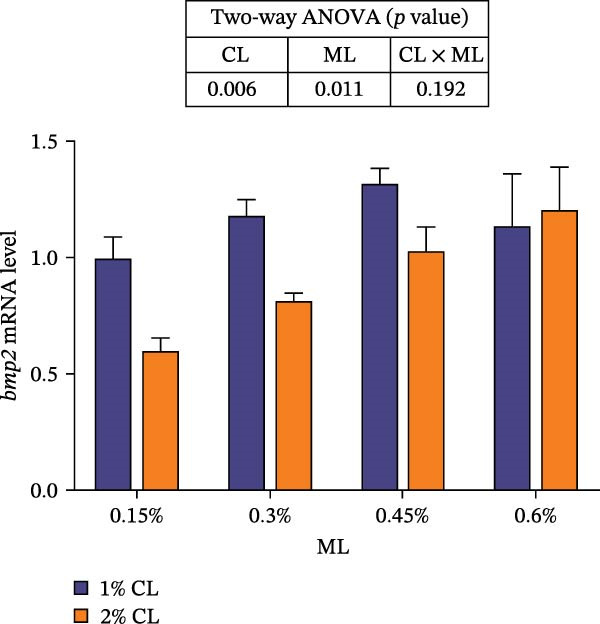
(D)

### 3.6. Molting‐Related Hormones Levels and Enzymes Activities

#### 3.6.1. The Levels of Molting‐Related Hormones in Hemolymph

As shown in Figure 5, high calcium significantly decreased the concentrations of ecdysone and MF at 0.15% magnesium level, while an opposite result was observed at higher magnesium levels (0.3% and 0.45% for ecdysone, 0.3% and 0.6% for MF) (*p* < 0.05). The concentrations of ecdysone and MF were both initially increased then decreased with rising magnesium levels (*p* < 0.05). Significant interactive effects between dietary calcium and magnesium levels were observed on the concentrations of ecdysone and MF (*p* < 0.001). At 1% calcium level, elevated magnesium levels decreased the concentration of ecdysone, while increased it at 2% calcium level.

Figure 5Effects of different dietary calcium and magnesium levels on the levels of molting‐related hormones in the hemolymph of *Eriocheir sinensis*. Different letters (lowercase for 1% calcium level and uppercase for 2% calcium level) indicate significant differences (*p* < 0.05) among different magnesium levels at the same calcium level.  ^∗^ indicates significant differences (*p* < 0.05) between different calcium levels at the same magnesium level.  ^∗^ *p* < 0.05,  ^∗∗^ *p* < 0.01,  ^∗∗∗^ *p* < 0.001, ns: no significant difference. (A) Ecdysone; (B) MF, methyl farnesoate. CL represents the calcium level, ML represents the magnesium level.

**Figure:**
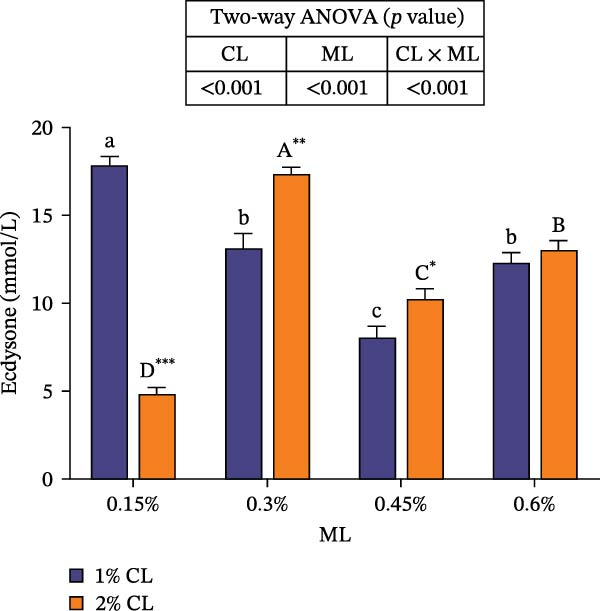
(A)

**Figure:**
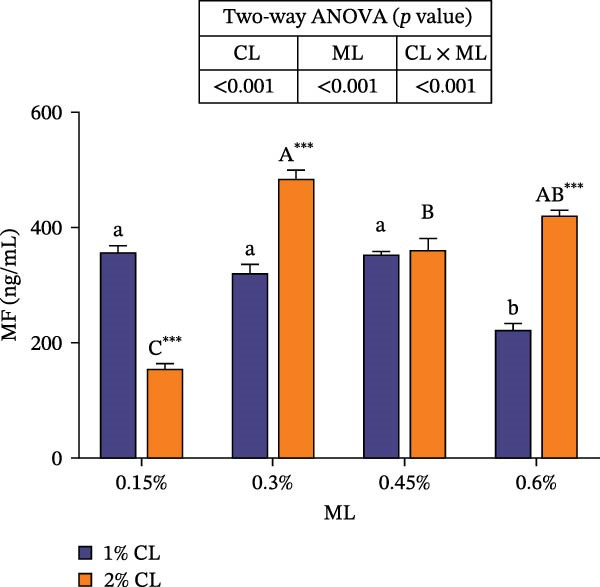
(B)

#### 3.6.2. The Activities of Epidermal Molting‐Related Enzymes

As presented in Figure 6, dietary calcium and magnesium levels had no significant effect on the activity of β‐NAG in the epidermis (*p* > 0.05). However, the activity of CHI initially increased then decreased with increasing magnesium levels (*p* < 0.05), reaching its maximum at 0.3% magnesium. A significant interactive effect was detected between calcium and magnesium levels in epidermal CHI activity (*p* < 0.05).

Figure 6Effects of different dietary calcium and magnesium levels on the activities of molting‐related enzymes in the epidermis of *Eriocheir sinensis*. Different uppercase letters indicate significant differences (*p* < 0.05) among different magnesium levels at 2% calcium.  ^∗^ indicates significant differences (*p* < 0.05) between different calcium levels at the same magnesium level.  ^∗^ *p* < 0.05, ns: no significant difference. (A) chi, chitinase; (B) β NAG, β‐N‐Acetylglucosaminidase. CL represents the calcium level, ML represents the magnesium level.

**Figure:**
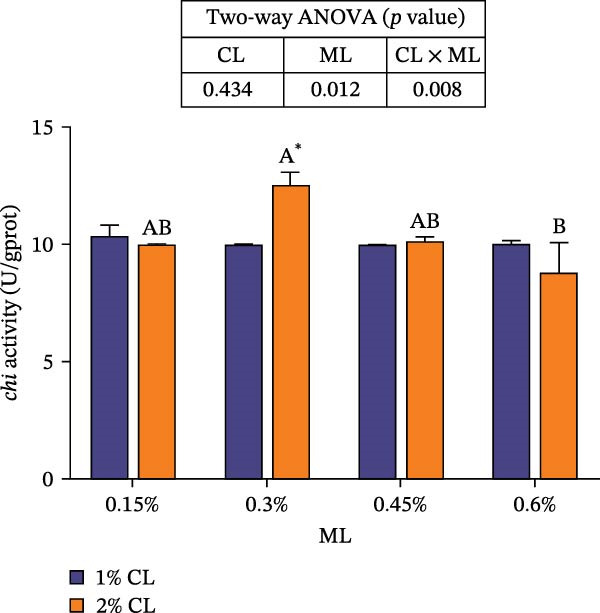
(A)

**Figure:**
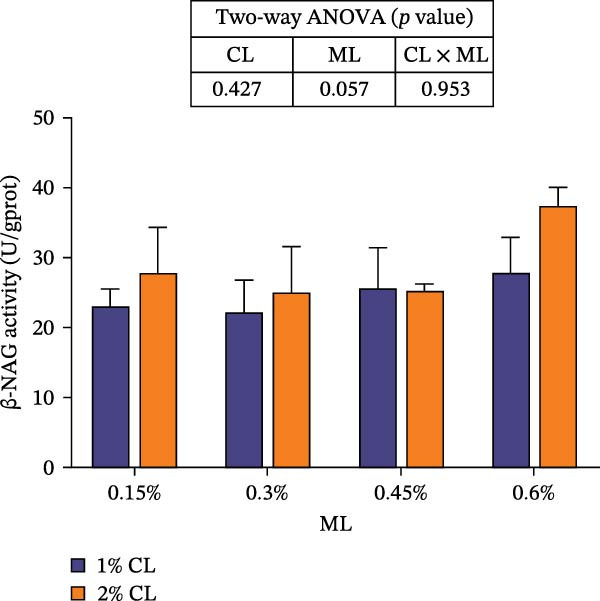
(B)

### 3.7. The Expressions of Molting‐Related Genes

As shown in Figure 7, the 2% calcium diet significantly decreased the expressions of retinoid X receptor (*rxr*) and molt‐inhibiting hormone (*mih*), but significantly increased the expression of ecdysone receptor (*ecr*) (*p*  < 0.05). Elevated magnesium levels led to a decreasing trend in expression of *rxr*, but an increasing trend in the expression of *mih* (*p*  < 0.05). However, the expressions of *chh* and *ecr* initially increased then decreased with increasing magnesium levels (*p*  < 0.05). Significant interactive effects were observed on the relative expressions of crustacean hyperglycemic hormone (*chh*), *ecr*, *rxr*, and *mih* (*p*  < 0.05). The expression of *ecr* achieved the highest value in the 2% calcium and 0.3% magnesium group, while the expression of mih achieved the lowest value.

Figure 7Effects of different dietary calcium and magnesium levels on the expression levels of molting‐related genes in the hepatopancreas of *Eriocheir sinensis*. Different letters (lowercase for 1% calcium level and uppercase for 2% calcium level) indicate significant differences (*p* < 0.05) among different magnesium levels at the same calcium level.  ^∗^ indicates significant differences (*p* < 0.05) between different calcium levels at the same magnesium level.  ^∗^ *p* < 0.05,  ^∗∗^ *p* < 0.01,  ^∗∗∗^ *p* < 0.001, ns: no significant difference. Data were normalized to the 1% Ca and 0.15% Mg group. (A) *c*hh, Crustacean hyperglycemic hormone; (B) *e*cr, Ecdysone receptor; (C) *r*xr, Retinoid X receptor; (D) *m*ih, Molt‐inhibiting hormone. CL represents the calcium level, ML represents the magnesium level.

**Figure:**
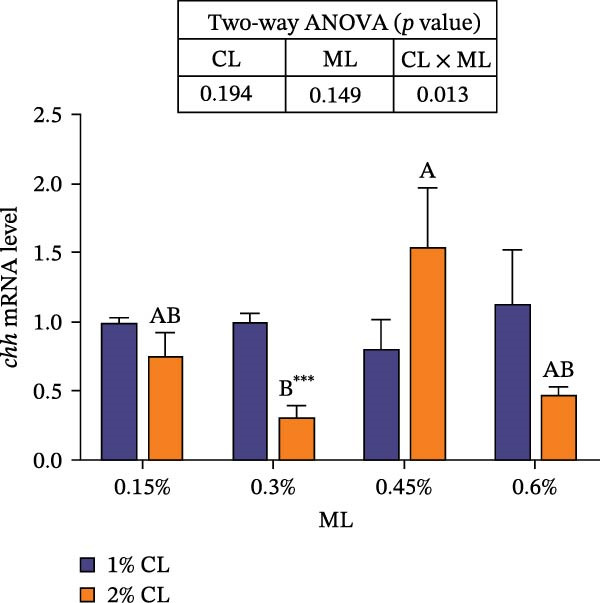
(A)

**Figure:**
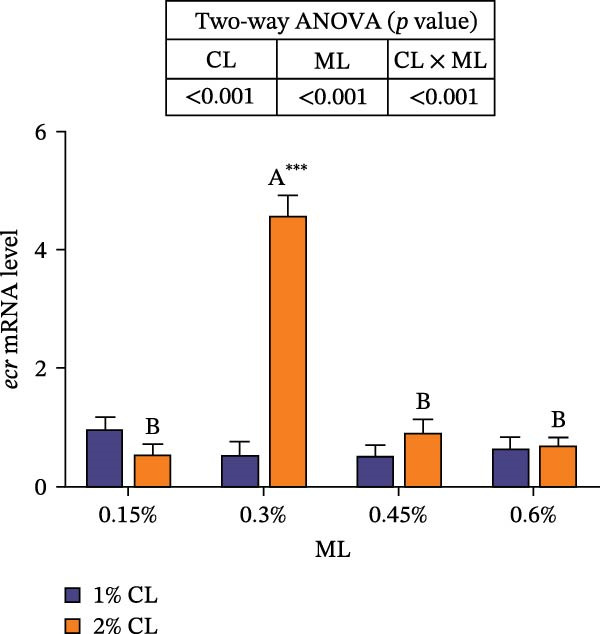
(B)

**Figure:**
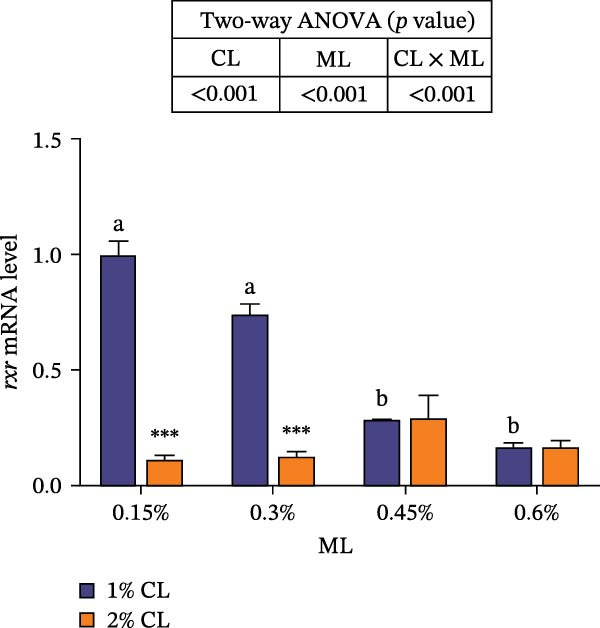
(C)

**Figure:**
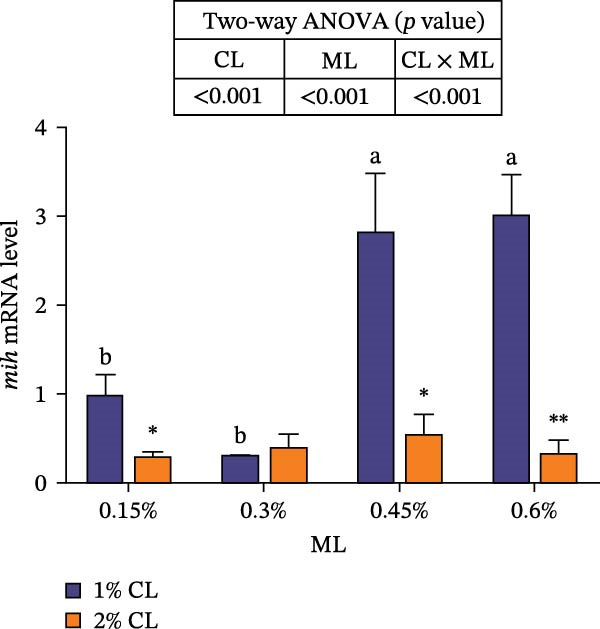
(D)

## 4. Discussion

In the present study, high calcium levels significantly reduced molting frequency. A similar result was also noted in WGR although no significant difference was noted. This suggests that excessive dietary calcium may inhibit the growth of *E. sinensis* potentially due to a long‐term disruption of the intracellular calcium homeostasis, leading to metabolic stress that suppresses growth. Additionally, high calcium levels may interfere with nutrient accumulation, such as lipids, resulting in a reduced HSI, as aligns with previous findings in the crayfish *Astacus leptodactylus* [7]. Furthermore, increasing dietary calcium levels significantly reduced carapace hardness, consistent with an earlier observation in aquatic environments [38], further supporting the notion that excessive calcium is detrimental to the growth of crab. When dietary magnesium levels increased from 0.15% to 0.6%, in the 1% calcium level groups, WGR and MR initial increased then decreased, although no statistical difference was noted in WGR and MR. It is hypothesized that an appropriate level of magnesium could facilitate protein, carbohydrate and lipid metabolism, thereby improving nutrient absorption and utilization, as consequently enhances growth performance [50, 51]. Notably, significant interactive effects between dietary calcium and magnesium levels were observed in WGR, MR, and carapace hardness. At 1% calcium, these parameters first increased then decreased with rising magnesium levels. In contrast, at 2% calcium, they showed an initial decline followed by an increase. This indicates that high dietary calcium levels may increase the dietary magnesium requirement of *E. sinensis*, likely because excess calcium inhibits magnesium absorption [52]. However, further studies should be conducted to validate this.

In the crustacean exoskeleton, collagen fibers are deposited in all layers except for the outermost epicuticle, contributing to exoskeletal hardness [53]. In the present study, increased dietary calcium levels significantly elevated the collagen fiber content in the carapace. Previous research has shown that high calcium concentrations can activate fibroblasts, thereby promoting collagen production [54]. However, the effect of dietary magnesium levels is more complex, since a significant interaction was observed between different dietary levels of calcium and magnesium in the relative collagen area in the carapace. At 1% calcium, increasing magnesium concentrations decreased the relative collagen area. However, an opposite result was observed when dietary calcium level reaches 2%. It is hypothesized that at the lower calcium level (1%), elevated magnesium competes with calcium for binding sites, reducing calcium absorption and subsequently impairing collagen fiber synthesis [31, 54]. When dietary calcium increased to 2%, the sufficient availability of calcium mitigates the competitive inhibition by magnesium. Moreover, magnesium may itself exert a synergistic or promotive effect on collagen formation. Thus, under high dietary calcium conditions, increasing magnesium levels significantly enhances collagen deposition in the carapace.

Prior to molting, *E. sinensis* reabsorbs mineral elements (such as calcium and magnesium) from the old exoskeleton, and temporarily stores them in tissues including the hemolymph and hepatopancreas for the subsequent mineralization of the new shell after ecdysis [55, 56]. In the present study, intestine and muscle calcium deposition decreased with increasing dietary calcium levels, while an opposite result was observed in whole‐body and exoskeletal calcium content. Generally, excessive calcium intake elevates blood calcium levels, thereby stimulating the release of calcitonin and parathyroid hormone, which can inhibit intestinal calcium absorption, and consequently reduce calcium accumulation in intestinal and muscular tissues [57]. In addition, over 80% of the total body calcium is deposited in the exoskeleton of crab [9]. When excess calcium is ingested, the crab appears to preferentially allocate calcium to the exoskeleton, enhancing its calcification and hardness—a phenomenon consistent with findings in cladocerans [58]. Magnesium serves as a cofactor for various ATPases [59]. At the 2% calcium level, increasing dietary magnesium levels enhanced Ca^2+^‐ATPase activity, facilitating greater calcium transport to the hepatopancreas, muscle, and exoskeleton, thereby increasing tissue calcium deposition. The transient receptor potential melastatin types 6 and 7 (TRPM6/7) play essential roles in the absorption and transport of calcium and magnesium ions, thereby maintaining intracellular and extracellular ion homeostasis [33]. In this study, the 2% calcium diet significantly reduced magnesium content in the hepatopancreas and muscle, likely because excess calcium competes with magnesium for binding sites on TRPM6/7 channels, thereby impairing magnesium absorption and reducing tissue magnesium levels. The hepatopancreas serves as a major mineral storage organ in crustaceans during the molting cycle [60]. Accordingly, as dietary magnesium levels increased, crabs accumulated surplus magnesium in the hepatopancreas, elevating its magnesium content. Furthermore, significant interactive effects between calcium and magnesium levels were observed on calcium content in the hepatopancreas, muscle, and exoskeleton, as well as on hepatopancreas magnesium content. At the 1% calcium level, increasing magnesium levels significantly raised hepatopancreas calcium content, while calcium levels in muscle and exoskeleton first decreased then increased. In contrast, at the 2% calcium level, increasing magnesium levels significantly increased calcium content in muscle and the exoskeleton. High dietary calcium levels have been reported to reduce vertebral calcium content in fish, thus impairing normal skeletal development [6], whereas high magnesium diets increase exoskeletal calcium content in Pacific white shrimp (*Litopenaeus vannamei*) [21]. Consistent with these observations, the present results suggest that under high dietary calcium conditions, *E. sinensis* may require additional magnesium to regulate tissue mineral deposition and ensure normal exoskeleton development.

To further investigate the transport and deposition patterns of calcium and magnesium in the body of *E. sinensis*, the expressions of genes related to calcium and magnesium absorption were determined in the hepatopancreas and intestine. The hepatopancreas is a vital organ for metabolism and mineral storage in crustaceans, where stored minerals (such as calcium and magnesium) facilitate the rapid mineralization of the exoskeleton during the post‐molt period [60]. Crustaceans primarily absorb calcium and magnesium ions from food through the intestine [61, 62]. In this study, elevated calcium levels significantly reduced the gene expressions of *crt*, *cnx*, and *nipa2* in the hepatopancreas. This indicated that under high‐calcium conditions, the crab may mitigate the detrimental effects of excessive calcium ions by downregulating the expressions of genes related to calcium absorption. This is supported by the facts that CRT and CNX are major calcium‐binding proteins in the endoplasmic reticulum, playing crucial roles in regulating intracellular homeostasis [63, 64]; while NIPA2 is a magnesium transporter located on the cell membrane, and is closely involved in regulating magnesium metabolism [65]. It is speculated that excess calcium may disrupt the calcium–magnesium balance in the body, inhibiting the expressions of related genes, consequently reducing calcium and magnesium content in the hepatopancreas. In contrast, an opposite result was noted in these genes in the intestine suggesting that high dietary calcium level activates the expressions of genes involved in calcium and magnesium absorption in the intestine, thereby promoting their uptake. This discrepancy may be due to the tissue‐specific functions in mineral utilization (e.g., storage and metabolism in the hepatopancreas and absorption in the intestine). When the calcium level was 1%, increased magnesium levels up‐regulated the expressions of *nipa2* in both the hepatopancreas and intestine. It is hypothesized that under high‐magnesium conditions, crabs may absorb more magnesium, leading to increased magnesium deposition in the hepatopancreas, thereby preparing for exoskeleton mineralization after molting. When the calcium level was 2%, increasing magnesium levels resulted in an initial decrease followed by an increase in the expressions of *crt* and *cnx* in the intestine. This indicates that under high‐calcium conditions, elevated magnesium levels may inhibit calcium absorption, as may be attributed to the competition for calcium‐binding sites. To maintain calcium balance, the expressions of *crt* and *cnx* is up‐regulated to promote intestinal calcium absorption [31]. Conversely, the expression of *nipa2* in the intestine was significantly reduced, likely as a mechanism to maintain magnesium homeostasis by down‐regulating magnesium absorption [65]. Furthermore, the interaction between different calcium and magnesium levels significantly affected the expressions of *crt*, *cnx*, and *nipa2* in both the hepatopancreas and intestine. An increase in magnesium levels did not affect the expression of *crt* in the 1% calcium groups, but up‐regulated its expression in the 2% calcium groups with the highest expression observed at a magnesium level of 0.6%. This suggests that under high dietary calcium levels are elevated, appropriately increasing magnesium levels may help crabs regulate the expressions of genes related to calcium and magnesium absorption, thereby maintaining calcium and magnesium homeostasis.

Molting in crustaceans is primarily regulated by molting hormone (ecdysone) and molt‐inhibiting hormone (MIH). Ecdysone is secreted by the Y‐organ and, upon entering the cell, binds to a heterodimeric complex composed of the ecdysone receptor (EcR) and the retinoid X receptor (RXR), activating the transcriptional expressions of downstream molting‐related genes [66, 67]. However, MIH and CHH regulate molting by inhibiting the secretion of ecdysone [68, 69]. In this study, elevated calcium levels significantly reduced the expressions of *chh*, *rxr*, and *mih*, but increased the expression of *ecr*, as well as the contents of ecdysone and MF. This suggests that high calcium ion concentrations may regulate the EcR/RXR complex by suppressing the expressions of *mih* and *chh*, while stimulating the secretion of ecdysone and MF. It is hypothesized that increased calcium ions may promote the formation of the EcR/RXR complex by activating the calcium/calmodulin‐dependent protein kinase pathway [70], thereby facilitating molting in crab. However, the influence of dietary magnesium levels is more complex, since an interaction between different dietary calcium and magnesium levels was observed in the hemolymph content of ecdysone and MF, as well as the gene expressions of *chh*, *ecr*, *rxr*, and *mih*. When dietary calcium level was 1%, increasing magnesium levels led to a significant decrease in the content of ecdysone and MF and the expression of *rxr*, while the expression of *mih* showed an opposite result. This result is justifiable because magnesium ions occupy calcium‐binding sites, reducing calcium influx and decreasing the activation of cyclic nucleotide phosphodiesterase, thereby upregulating *mih* expression and inhibiting the secretion of molting‐related hormones [71]. When dietary calcium level was 2%, increasing magnesium levels caused a first increase then a decrease in the concentrations of ecdysone and MF and the expression of *ecr*, while the expression of *chh* exhibited the opposite trend. This may be due to the fact that, under high dietary calcium conditions, an appropriate increase of magnesium facilitates the competition with calcium for binding sites, reducing calcium influx [31], as helps maintain intracellular calcium homeostasis, and allows the crab to allocate more energy to molting. However, with a further increase in magnesium levels, more binding sites are occupied by magnesium, leading to reduced intracellular calcium levels, which subsequently inhibits molting [72]. These findings imply that more dietary magnesium is needed by crabs to maintain the normal molting activity when fed high‐calcium diets.

The molting process of the *E. sinensis* involves the shedding of the old exoskeleton and the formation of a new one. Molting hormone is closely associated with the expression of the *chi* gene [73]. In this study, elevated dietary calcium levels significantly increased CHI activity and the expressions of *chi* and *cpcbm*, but significantly decreased the expression of *chs*. The change in *chi* expression was consistent with the variation in molting hormone content, indicating that high dietary calcium levels promote molting capacity in the crab. Chitin is a major component of the crab exoskeleton, and its degradation is regulated by CHI and β‐NAG secreted from the epidermal tissue [74, 75]. During molting, crustaceans require chitinolytic enzymes to break down chitin in the old exoskeleton to facilitate ecdysis [76], while CHS is a key enzyme responsible for chitin synthesis [77]. It is hypothesized that high dietary calcium levels may promote the secretion of molting hormones, thereby upregulating *chi* expression, enhancing CHI activity, and simultaneously inhibiting chitin synthesis, as facilitates the shedding of the old exoskeleton. In addition, the upregulation of *cpcbm* expression also suggests that higher calcium levels facilitate the formation of new exoskeleton during post‐molt [47]. When the calcium level was 2%, increasing magnesium levels led to an initial increase followed by a decrease in CHI activity, a trend consistent with that of molting hormone. Although the change in *chi* expression was not statistically significant, it showed a similar pattern, possibly indicating that magnesium regulates CHI activity via its effect on molting hormone [73]. Elevated magnesium may compete with calcium for binding sites, reducing calcium influx [31], thereby downregulating *cpcbm* expression. Furthermore, significant interactive effects between dietary calcium and magnesium levels were observed in CHI activity and the expressions of *chi*, *chs*, and *cpcbm*. In the 1% calcium groups, *chi* expression was highest at a magnesium level of 0.15%. In contrast, in the 2% calcium groups, *chi* expression peaked at the 0.3% magnesium group, where *chs* expression was the lowest. This suggests that a magnesium level of 0.3% is more suitable under high calcium conditions, whereas 0.15% magnesium is sufficient for molting requirements under normal calcium levels, consistent with changes in molting‐related hormones. These results indicate that increasing dietary magnesium appropriately may improve the molting performance of crabs offered high‐calcium diets. Nevertheless, further research is needed to elucidate the underlying mechanisms.

## 5. Conclusion

In summary, the present study demonstrated that dietary calcium and magnesium levels interactively influence the growth performance, tissue calcium and magnesium deposition, exoskeleton development, and molting success of *E. sinensis*. While increasing dietary calcium levels to some extent promotes molting, it also disrupts the calcium homeostasis, ultimately impairing growth. Based on growth performance, 0.3% and 0.6% magnesium levels (analyzed values: 0.41% and 0.69%) are recommended when dietary calcium levels are 1% and 2%, respectively.

## Author Contributions

Han Chen, Xiangfei Li, and Wenbin Liu designed the study. Han Chen conducted the experiments, analyzed the data, and wrote the original manuscript. Wenbin Liu performed the conceptualization, manuscript editing, funding acquisition and supervision. Yueyun Guo conducted the experiments and contributed reagents. Sisi Xiong contributed reagents and materials. Zishang Liu contributed reagents. Yanzou Dong polished the manuscript. Beile Ye provided the software. Lei Xu provided feed ingredients and premix. Pan Wang provided feed ingredients and premix. Xiangfei Li performed the experimental guidance, funding acquisition, and supervision.

## Funding

This research was supported by the Jiangsu Agricultural Science and Technology Independent Innovation Fund (Grant Number CX(24)1013) and the Fund of Fujian Key Laboratory of Functional Aquafeed and Culture Environment Control (FACE20230006).

## Conflicts of Interest

The authors declare no conflicts of interest.

## Data Availability

The data that support the findings of this study are available from the corresponding author upon reasonable request.
